# Utilizing %Carbohydrate-deficient Transferrin as a Biomarker to Complement Interviews in Stratifying Alcohol Consumption in Patients with Alcohol Dependence: Aiming for Application to Fatty Liver Disease

**DOI:** 10.31662/jmaj.2025-0109

**Published:** 2025-06-13

**Authors:** Motoh Iwasa, Akiko Eguchi, Tatsuya Suzuki, Ryuta Shigefuku, Saeko Nagao, Masayuki Morikawa, Kazushi Sugimoto, Hayato Nakagawa

**Affiliations:** 1 Department of Gastroenterology and Hepatology, Mie University Graduate School of Medicine, Mie, Japan; 2 Department of Gastroenterology, Murase Hospital, Mie, Japan; 3 Biobank Center, Mie University Hospital, Mie, Japan; 4 Department of Gastroenterology and Hepatology, St. Marianna University School of Medicine, Kanagawa, Japan; 5 Kure Midorigaoka Hospital, Hiroshima, Japan; 6 Mie Prefectural Mental Care Center, Mie, Japan; 7 Department of Clinical Laboratory, Mie University Hospital, Mie, Japan

**Keywords:** alcohol-associated/related liver disease (ALD), alcohol dependence, carbohydrate-deficient transferrin (CDT), biomarker, steatotic liver disease

## Abstract

**Introduction:**

Alcohol dependence is linked to various issues, including not only alcohol-associated/related liver disease (ALD) but also social isolation, making the assessment of alcohol consumption crucial for patient management. Meanwhile, a multisociety consensus group has introduced a new classification for steatotic liver disease (SLD), including ALD, based on alcohol consumption. The evaluation of alcohol intake uses tools such as the Alcohol Use Disorders Identification Test and Lifetime Drinking History; however, these tools may lack accuracy in clinical settings. Carbohydrate-deficient transferrin (%CDT) is a quantitative and objective biomarker for alcohol consumption. Therefore, we aimed to determine %CDT values that stratify alcohol consumption.

**Methods:**

This cross-sectional analysis included 285 serum samples from patients receiving inpatient or outpatient treatment at two specialized alcohol dependency medical centers. Participants were alcohol-dependent patients who underwent detailed interviews regarding alcohol consumption, biochemical blood tests, and %CDT testing.

**Results:**

Among the 285 samples, 32.6%, 19.6%, and 47.7% corresponded to alcohol consumption levels of ≤30 g/day for men/≤20 g/day for women, 30-60 g/day for men/20-50 g/day for women, and ≥60 g/day for men/≥50 g/day for women, respectively. %CDT values increased with increasing alcohol consumption (p < 0.05-0.0001). The cutoff values reflecting alcohol consumption of 30 g/day for men/20 g/day for women and 60g/day for men/50g/day for women were 1.67% and 2.48%, respectively. Gamma-glutamyl transferase (GGT) and GGT-CDT were able to distinguish between alcohol consumption above and below 60 g/day for men and 50 g/day for women (p < 0.0001). However, they had difficulty distinguishing between alcohol consumption above and below 30 g/day for men and 20 g/day for women.

**Conclusions:**

%CDT, in conjunction with detailed interviews, can be used to detect alcohol consumption, particularly to distinguish whether it exceeds 30 g/day in men and 20 g/day in women. Applying this to the clinical management of patients with alcohol dependence accompanied by ALD or SLD may contribute to improving the quality of care.

## Introduction

According to a report by the World Health Organization, alcohol dependence has become one of the five major risk factors for global morbidity, disability, and mortality. It can cause severe damage to the liver and brain, increase the risk of domestic violence and traffic accidents, reduce employment capacity, and lead to social isolation, making early treatment and support crucial ^(1)^. In recent years, steatotic liver disease (SLD) has been used as an umbrella term, including alcohol-associated/related liver disease (ALD). SLD is classified based on the following alcohol consumption levels: ≤30 g/day for men/≤20 g/day for women, 30-60 g/day for men/20-50 g/day for women, ≥60 g/day for men/≥50 g/day for women ^(2)^.

The evaluation of alcohol consumption uses tools such as the Alcohol Use Disorders Identification Test (AUDIT) ^(3)^ and Lifetime Drinking History (LDH) ^(4)^, but these methods have limitations. Patients often underestimate their alcohol intake, hindering accurate evaluation. Furthermore, these tools struggle to account for both the frequency of drinking and occasional binge drinking ^(5)^. Based on these factors, there is a need for objective biomarkers to assess alcohol consumption. In alcohol dependence, biomarkers are used not only as an aid to diagnosis but also to support recovery and serve as catalysts for discussion with the patient. While biochemical indicators of chronic drinking, such as elevated serum gamma-glutamyl transferase (GGT), an aspartate aminotransferase (AST)/alanine aminotransferase (ALT) ratio >1, changes in red blood cell membrane lipid composition leading to increased mean corpuscular volume (MCV), and elevated immunoglobulin A, can be helpful, accurately estimating alcohol consumption with these markers alone remains challenging. It has been reported that exceeding 60g of alcohol per day for more than two weeks leads to sustained inhibition of glycosylation in the Golgi body, resulting in the production of carbohydrate-deficient transferrin (CDT). Given CDT’s half-life of approximately 15 days, it is a useful marker for estimating recent alcohol consumption ^(6), (7)^. Therefore, this study aimed to determine the %CDT values that stratify alcohol consumption levels.

## Materials and Methods

### Study subjects

This study was an observational study conducted at two psychiatric hospitals specializing in alcohol dependence: the Mie Prefectural Mental Care Center and the Kure Midorigaoka Hospital. It was conducted in accordance with the provisions of the 1975 Declaration of Helsinki and was approved by the Clinical Research Ethics Review Committee of Mie University Hospital. Written informed consent was obtained from participants prior to participation. The inclusion criteria were: patients aged 20 years or older, diagnosed with alcohol dependence by a psychiatrist at one of the two specialized medical institutions. The diagnosis of alcohol dependence was initially screened using the AUDIT-10 ^(3)^ or the Kurume style alcohol dependence screening test ^(8)^ by the psychiatrist or relevant personnel, as appropriate, and was ultimately confirmed by the psychiatrist based on the 10th edition of the International Classification of Diseases. All participants were Japanese. Ultrasound or computed tomography scans, performed by the referring physician or psychiatric hospitals, were used to diagnose SLD based on positive hepatorenal contrast or decreased liver computed tomography density. The exclusion criteria were: patients with liver diseases (including hepatitis B, hepatitis C, and autoimmune hepatitis), malignant tumors, end-stage liver and/or kidney failure, and other serious physical illnesses accompanied by other mental or cognitive disorders.

### Data collection and clinical evaluation

The patient cohort consisted of individuals diagnosed with alcohol dependence and a history of problematic alcohol consumption exceeding one year. This group included individuals who engaged in treatment with their attending physician and achieved abstinence or reduced alcohol intake, as well as those who initially abstained but subsequently relapsed. Information on alcohol consumption at the time of the visit was collected by the psychiatrist in the examination room on the same day as blood sampling, including %CDT. Patients were interviewed about their abstinence status over the past 30 days. For those who reported alcohol consumption, daily intake was recorded as the number of drinks (1 drink = 10 g of pure alcohol) or in grams of alcohol and used to calculate the average daily consumption (g/day) over the past 30 days. For hospitalized patients, alcohol consumption was assumed to be absent. Based on alcohol intake over the past 30 days, each patient was classified into the following three categories according to the new SLD diagnostic criteria ^(2)^: i) alcohol consumption levels of ≤30 g/day for men/≤20 g/day for women (small amount or less drinking group), ii) alcohol consumption levels of 30-60 g/day for men/20-50 g/day for women (moderate drinking group), iii) alcohol consumption levels of ≥60 g/day for men/≥50 g/day for women (excessive drinking group). Body mass index is calculated by dividing body weight (kg) by the square of height (m). Blood samples collected from the same patient were used to investigate whether %CDT could be used as an indicator of alcohol intake. AST, ALT, GGT, MCV, total bilirubin (Bil), and albumin were measured at each hospital. Isolated serum samples were stored at −80°C until %CDT measurement was performed. Serum %CDT levels were analyzed using the N Latex CDT direct immune turbidimetric method (BNProSpec S/N:171885; Siemens Healthcare Diagnostics, Kawasaki, Japan). The albumin-bilirubin (ALBI) score was calculated using serum albumin and Bil ^(9)^. The fibrosis-4 (FIB-4) index was calculated based on age, AST, ALT, and platelet count ^(10)^. Additionally, the GGT-CDT value was calculated using the formula proposed in previous studies: 0.8 × ln(GGT) + 1.3 × ln(%CDT) ^(11)^.

### Statistical analysis

Continuous variables are presented as mean ± standard deviation, and categorical variables are shown as numbers and frequencies (percentages) of patients. Analysis of variance was used for comparing values across the three categories. The male-to-female ratio was tested using a contingency table. Receiver operator characteristic (ROC) curves and the corresponding area under the curve (AUC) were used to obtain cutoffs estimating each serum marker’s ability to calculate alcohol consumption within the past 30 days. The Youden index was applied to calculate the optimal cutoff point. Outlier testing was not performed, and all values were retained for analysis. The statistical analyses were performed using Graph Pad (Graph Pad Software Inc., San Diego, CA) for comparison of continuous variables. Significant differences were considered at p < 0.05.

## Results

### Clinical characteristics of the study cohort classified by alcohol consumption

A total of 285 serum samples collected from two specialized alcohol dependency medical centers were analyzed. Patient characteristics are summarized in Table 1. Of these samples, 32.6%, 19.6%, and 47.7% corresponded to the small amount or less drinking group, the moderate drinking group, and the excessive drinking group, respectively. The low alcohol consumption group had a significantly higher proportion of older women (p < 0.001). AST, ALT, and GGT levels increased in parallel with alcohol consumption, while MCV values showed no significant differences. The average body mass index was below 25 kg/m^2^, with few participants classified as obese. The FIB-4 index was >3.25 in 40 samples, ≤3.25 in 243 samples, and unknown in two samples, indicating advanced liver fibrosis in 14% of cases ^(12)^. Additionally, based on Bil levels and ALBI scores, a few cases exhibited reduced hepatic functional reserve.

**Table 1:** Patient Characteristics.

	Alcohol consumption levels		
Characteristics	Small amount or less	Moderate	Excessive
	(≤30 g/day for men	30-60 g/day for men	≤60 g/day for men
	≤20 g/day for women	20-50 g/day for women	≤50 g/day for women)
Age (years)	58 ± 13 (a, b,^****^)	51 ± 13	51 ± 13
Male sex, n (%)	83, 89 (a, b,^****^)	39, 70	98, 72
BMI (kg/m^2^)	23.0 ± 4.2	22.3 ± 3.3	22.5 ± 3.9
AST (U/L)	30.5 ± 22.6 (b^***^)	35.7 ± 34.8 (c^****^)	58.6 ± 79.2
ALT (U/L)	14.4 ± 25.5 (a, b^****^)	28.6 ± 28.4 (c^**^)	41.0 ± 39.8
GGT (U/L)	93.5 ± 156.0	102.8 ± 189.7	219.2 ± 311.3
Bil (U/L)	0.74 ± 0.44	0.74 ± 0.79	0.83 ± 0.69
MCV (fL)	94.8 ± 5.5	94.7 ± 6.6	96.0 ± 7.1
CDT (%)	1.95 ± 1.12 (a^*^, b^****^)	2.44 ± 1.56	2.68 ± 1.37
GGT-CDT	3.97 ± 0.91 (b^****^)	4.13 ± 1.20 (c^***^)	4.86 ± 1.33
ALBI score	-2.82 ± 0.46	-2.87 ± 0.56 (c^*^)	-2.70 ± 0.49
FIB-4 index	1.59 ± 1.02	2.05 ± 2.76 (c^*^)	2.58 ± 2.65

ALBI: albumin-bilirubin; ALT: alanine aminotransferase; AST: aspartate aminotransferase; Bil: total bilirubin; CDT: carbohydrate-deficient transferrin; FIB-4: fibrosis-4; GGT: gamma-glutamyl transpeptidase; MCV: mean corpuscular volume; SD: standard deviation. Values are mean ± SD. a: Small amount or less vs. moderate, b: Small amount or less vs. excessive, c: Moderate vs. excessive. ^*^: p < 0.05; ^**^: p < 0.01; ^***^: p < 0.001; ^****^: p < 0.0001

### Relationship between %CDT and alcohol consumption, and its ability to predict alcohol intake

**Figure 1.**
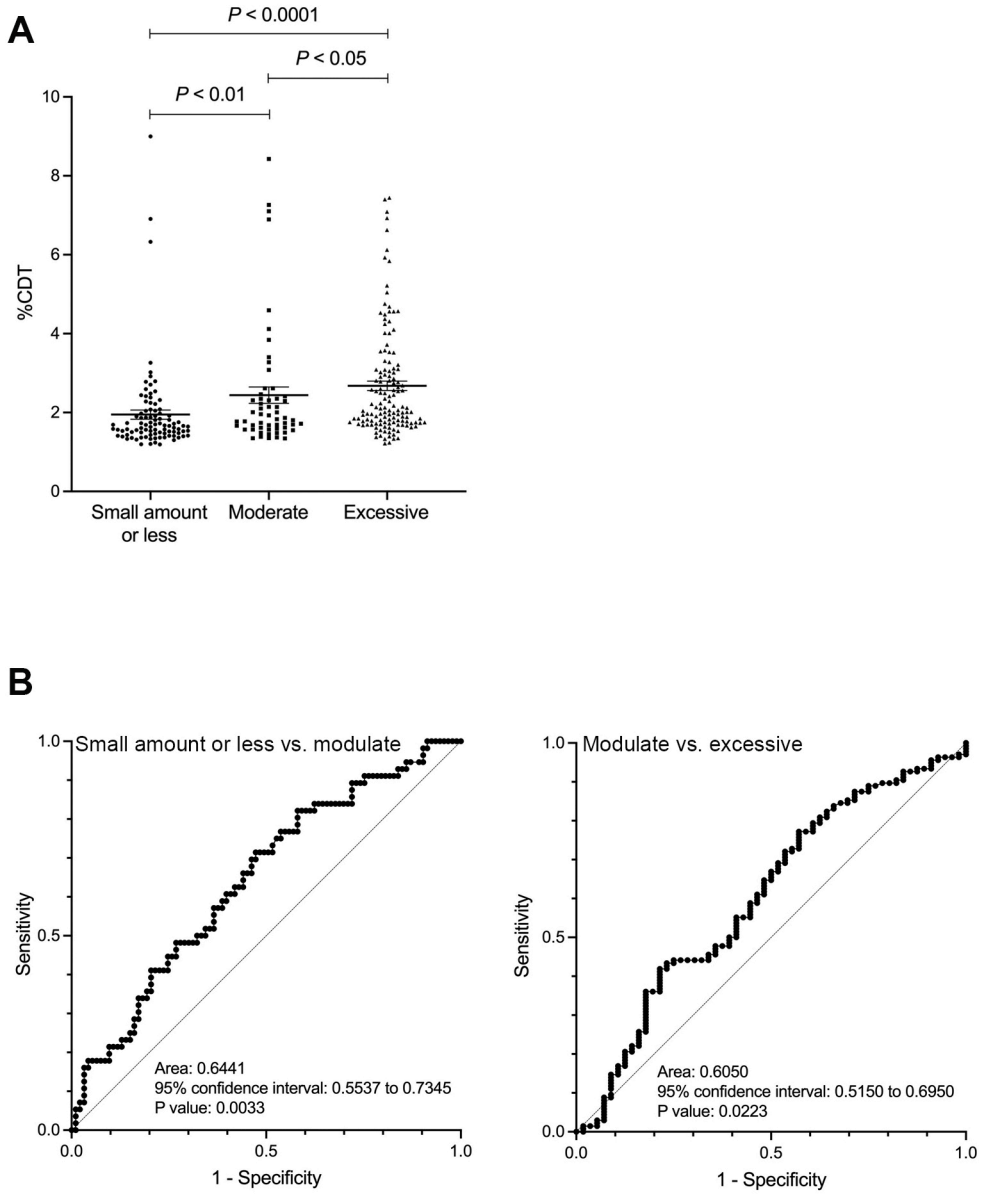
A: The relationship between alcohol consumption and %CDT values. B: ROC curve for distinguishing between small amount or less and moderate drinking groups, and between moderate and excessive drinking groups using CDT. CDT: carbohydrate-deficient transferrin; ROC: receiver operator characteristic.

The %CDT value gradually increased with the amount of alcohol intake, rising from light to moderate and excessive consumption (comparison between the small amount or less drinking group and the moderate drinking group: p < 0.01; comparison between the moderate and excessive drinking groups: p < 0.05) (Figure 1A). ROC analysis of the ability of %CDT to discriminate between the light and moderate drinking groups showed an AUC of 0.644 (p < 0.01), while its ability to discriminate between the moderate and excessive drinking groups showed an AUC of 0.605 (p < 0.05) (Figure 1B). The cutoff values corresponding to alcohol consumption of 30 g/day for men and 20 g/day for women, and 60 g/day for men and 50 g/day for women, were 1.67% (sensitivity 71.4%, specificity 52.7%, positive predictive value (PPV) 47.0%, and negative predictive value (NPV) 74.2%) and 2.48% (sensitivity 41.9%, specificity 78.4%, PPV 82.6%, and NPV 35.8%), respectively. The %CDT value of 1.93% was also extracted as the cutoff corresponding to 30 g/day for men and 20 g/day for women. Using this cutoff, sensitivity was 48.2%, specificity was 73.1%, PPV was 51.0%, and NPV was 74.2%.

### Relationship between GGT and GGT-CDT with alcohol consumption and their ability to predict alcohol intake

**Figure 2.**
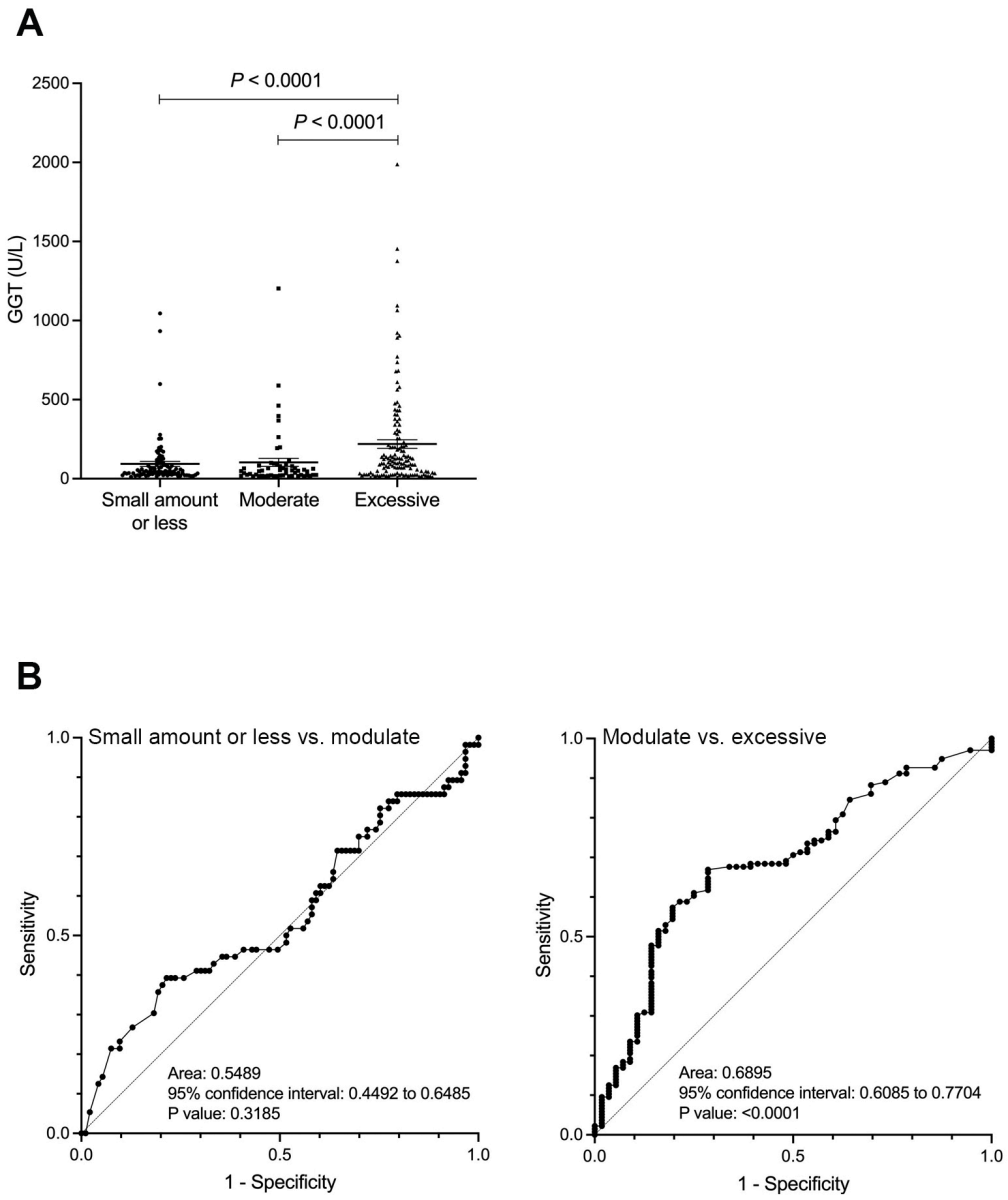
A: The relationship between alcohol consumption and GGT values. B: ROC curve for distinguishing between small amount or less and moderate drinking groups, and between moderate and excessive drinking groups using GGT. GGT: gamma-glutamyl transferase; ROC: receiver operator characteristic.

**Figure 3.**
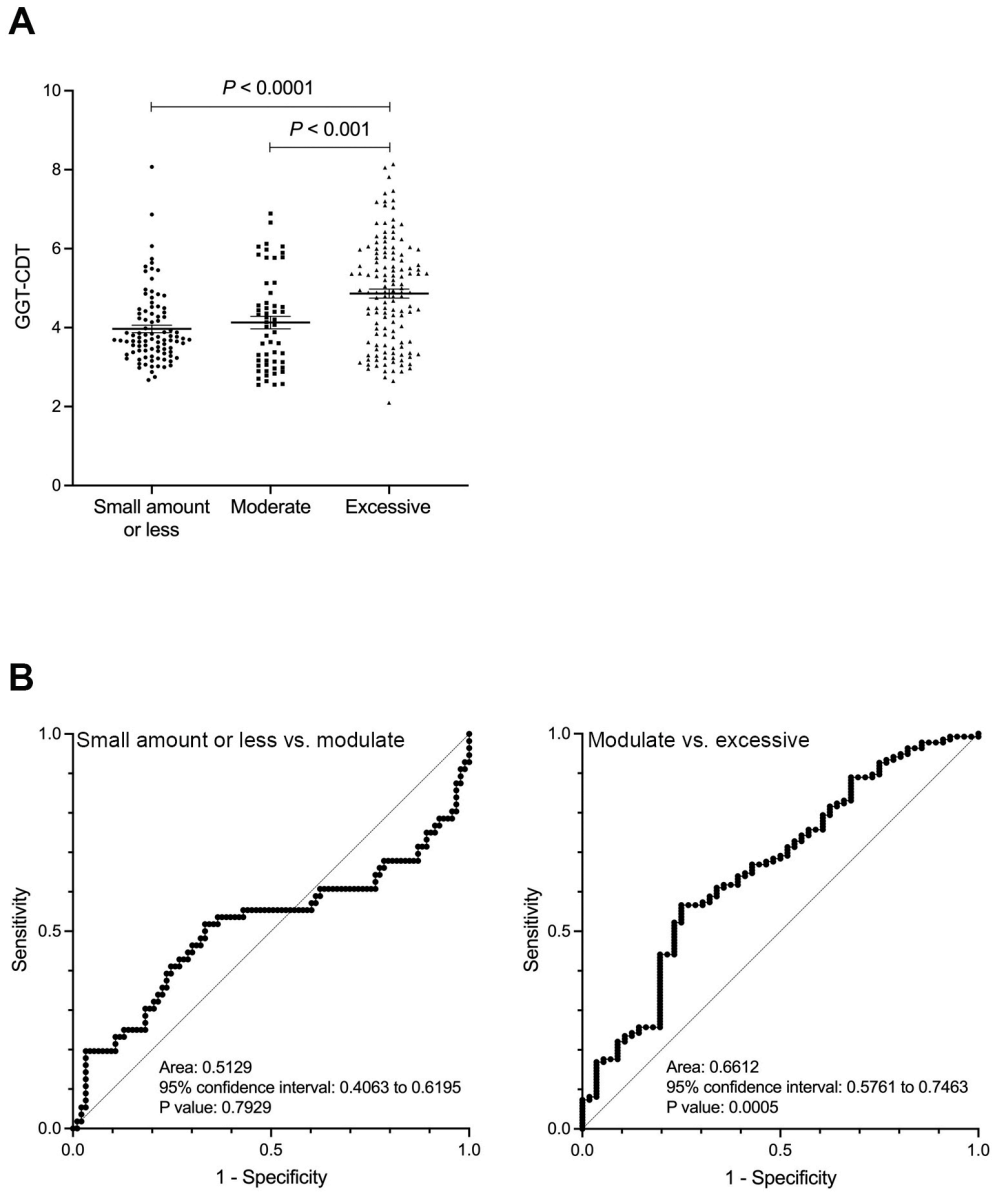
A: The relationship between alcohol consumption and GGT-CDT values. B: ROC curve for distinguishing between small amount or less and moderate drinking groups, and between moderate and excessive drinking groups using GGT-CDT. CDT: carbohydrate-deficient transferrin; GGT: gamma-glutamyl transferase; ROC: receiver operator characteristic.

In contrast, the GGT and GGT-CDT values showed a significant difference between the moderate and excessive drinking groups (p < 0.0001), but no significant difference was observed between the small amount or less drinking group and the moderate drinking group (Figure 2A and 3A). Similarly, ROC analysis for discriminating between the moderate and excessive drinking groups showed a favorable AUC of 0.690 and 0.661 (p < 0.001), whereas discrimination between the small amount or less drinking group and the moderate drinking group was not significant (p = 0.319, p = 0.793) (Figure 2B and 3B). The cutoff values corresponding to alcohol consumption of 30 g/day for men and 20 g/day for women, and 60 g/day for men and 50 g/day for women, were 26 U/L (sensitivity 39.3%, specificity 78.5%, PPV 31.8%, and NPV 47.6%) and 68 U/L (sensitivity 66.9%, specificity 71.4%, PPV 85.0%, and NPV 47.1%) for GGT, and 4 (sensitivity 51.8%, specificity 66.7%, PPV 48.3%, and NPV 69.7%) and 4.64 (sensitivity 56.6%, specificity 75.0%, PPV 84.6%, and NPV 41.6%) for GGT-CDT, respectively.

## Discussion

Clinical issues in alcohol dependence include the challenge of accurately assessing a patient’s alcohol consumption, which can lead to errors in treatment planning and prognosis. To address these issues, the use of objective biomarkers that reflect alcohol consumption is essential. Biomarkers serve not only as aids in diagnosis but also in supporting recovery and facilitating discussions with the patient, helping to monitor treatment effectiveness and adjust therapeutic strategies. Key principles for their use include discussing biomarker testing with patients beforehand to maintain the therapeutic alliance and encourage more accurate disclosure of alcohol use ^(13)^. This approach enables the provision of more effective and personalized treatment. Meanwhile, current SLD treatment involves classifying patients into categories such as ALD based on the presence or absence of metabolic abnormalities and alcohol intake, to estimate their pathophysiology and prognosis, thereby guiding treatment strategies ^(14)^. Consequently, the importance of accurately assessing alcohol consumption in SLD management is increasing ^(15)^. Current assessment methods, such as the AUDIT ^(3)^ and LDH ^(4)^, are limited by issues such as patients underestimating their alcohol intake, making accurate evaluation difficult, and the challenge of accounting for both drinking frequency and occasional binge drinking ^(5)^. Indeed, in this study, we observed many patients with elevated GGT and %CDT levels despite self-reporting abstinence. Therefore, we stratified self-reported alcohol intake and compared it with measured CDT values to investigate their predictive performance. Our results demonstrated a moderate correlation between self-reported alcohol intake and CDT levels, with CDT showing somewhat better predictive performance compared to GGT and the GGT-CDT combination. However, since GGT is influenced by alcohol consumption exceeding 30 days prior and MCV reflects even earlier consumption patterns, these markers may not accurately assess current alcohol intake in individuals with extreme drinking habits, such as those with alcohol dependence. Consequently, further investigation in a separate cohort is warranted to address this matter.

Several studies have reported the diagnostic performance of %CDT ^(16), (17), (18), (19), (20)^; however, the diagnostic sensitivity and cutoff values for %CDT show considerable variability due to differences in study populations and the criteria used to evaluate alcohol consumption ^(17), (21)^. Published reports indicate that the overall mean diagnostic sensitivity of %CDT ranges from 50% to 70%, with values varying from very low (<40%) to very high (>90%) ^(17), (18), (19), (20)^. Furthermore, the reported cutoff values for %CDT vary widely, ranging from 1.2% to 3.0% ^(17), (18), (19), (20)^. In the present study, the cutoff values reflecting alcohol consumption of 30 g/day for men and 20 g/day for women, as well as 60 g/day for men and 50 g/day for women, were identified as 1.67% and 2.48%, respectively. Furthermore, the %CDT value of 1.93% was also extracted as the cutoff for small amounts or less drinking; however, compared to 1.67%, this value showed an improvement in specificity, while sensitivity decreased. Since %CDT values are affected by race ^(22)^, determining a %CDT cutoff value for Japanese patients is critically important for clinicians. We believe that objective indicators are necessary to distinguish alcohol consumption levels of ≤30 g/day for men and ≤20 g/day for women, 30-60 g/day for men and 20-50 g/day for women, and ≥60 g/day for men and ≥50 g/day for women, as this may improve the quality of care for alcohol dependence. Additionally, to apply the current cutoff values to SLD patients, further validation in a separate cohort is required.

Recently, Hansen et al. ^(23)^ compared the use of interviews, phosphatidylethanol (PEth), a more direct biomarker of alcohol consumption, and %CDT in the context of metabolic dysfunction-associated SLD (MASLD) with increased alcohol intake (MetALD) and ALD. Alcohol consumption was assessed through interviews, showing a strong correlation with PEth (r = 0.617) and a moderate correlation with %CDT (r = 0.316). However, PEth outperformed interviews in predicting liver failure and mortality ^(23)^. In this study, the ability of %CDT to distinguish alcohol consumption levels was not sufficiently satisfactory. PEth may be superior to CDT, and it is necessary to investigate the usefulness of PEth, which has been reported to be effective overseas, in Japan as well.

A major strength of this study is the cohort consisting of a large number of patients strictly managed by specialists in alcohol dependence, as well as the measurement of %CDT using blood samples collected on the same day as the self-reported data on alcohol use.

The limitations of this study are as follows: it aims to determine %CDT values that reflect self-reported alcohol intake (noting that self-reported intake may differ from actual alcohol consumption); the study targets patients with alcohol dependence, who are significantly different from the general population in Japan ^(24), (25)^; multiple data points are used from the same patients; it cannot measure PEth ^(26)^; and it is a study targeting only Japanese individuals.

### Conclusions

%CDT, in conjunction with detailed interviews, can be used to detect alcohol consumption in SLD, particularly to distinguish whether it exceeds 30 g/day in men and 20 g/day in women. Applying this to the clinical management of patients with alcohol dependence accompanied by ALD or SLD may contribute to improving the quality of care. Therefore, further studies are needed to validate these findings in other cohorts and clinical settings, with the hope of also demonstrating the utility of %CDT in MASLD and MetALD in the future.

## Article Information

### Conflicts of Interest

None

### Acknowledgement

The authors sincerely thank all of the medical staff for data collection, especially Mr. Mizuki Tanaka, Ms. Masako Nishimoto (Kure Midorigaoka Hospital), and Ms. Tomoko Obe (Mie Prefectural Mental Care Center).

### Author Contributions

Conception and design of study: Motoh Iwasa and Akiko Eguchi; Acquisition of data: Tatsuya Suzuki, Ryuta Shigefuku, Saeko Nagao, Masayuki Morikawa, Kazushi Sugimoto; Interpretation of data: Hayato Nakagawa; Drafting of the manuscript: Motoh Iwasa. All authors reviewed and approved the manuscript.
